# Analectic

**Published:** 1834

**Authors:** 


### ANALECTIC, ANALYTICAL, AND ORIGINAL.

### I. Analectic.

*Ad Sylvas Nuncius.*

1. *On Hysteralgia, or Irritable Uterus. By Dr. D. Davis.*—In that very excellent work now publishing in parts by Dr. Davis, we find an article on hysteralgia, which we deem worthy of notice. The term was first given to the complaint by the late Dr. Gooch, having previously gone under the name of "painful menstruation"—"uterine irritation"—"chronic inflammation of the uterus," &c. Dr. Gooch's description of the hysteralgia is adopted by our author, who next proceeds to criticise the theory of Dr. Gooch, who considered hysteralgia as a purely functional disorder, unconnected with inflammation, and not ending in any change of structure.

"This plausible theory, (says Dr. D.,) of an exquisitely painful disease, without the co-existence of inflammatory action of the affected organ, must at best, in the present state of our knowledge, be considered doubtful as to its correctness. It is not even certain that we are yet acquainted with all the possible forms of inflammation, so as to be competent to assert broadly and emphatically that this or that variety of inflammation should have a natural and necessary tendency to end in disorganization of structure. It is not easy to conceive of certain forms of rheumatalgic affections, for example, such as lumbago and sciatica, without connecting with them the idea of an inflammatory condition of the tissues principally concerned; and yet on that account, who ever supposes that such inflammatory actions have a natural and necessary tendency to end in malignant disorganization of structure? If muscular fibres be a constituent tissue of the uterus, why not such fibres become the subjects of a painful inflammatory affection, a truly rheumatalgic affection, without being followed, any more than in the other case, by a malignant disorganization of structure? It is well known that the uterus is not unfrequently the subject of very painful states, occasioned exclusively by functional causes, as we see constantly exemplified in cases of disordered menstruation, leucorrhœa, etc.; but does it necessarily follow, that such morbid conditions are essentially independent of all inflammatory action? Or rather, is it not demonstrable that of some of them, at all events, inflammatory action is an essential attribute? And yet we find that such painful states, such demonstrably inflammatory affections, may be sustained for many years without producing malignant disorganization of structure. The limits subsisting between the phenomena respectively of irritation and inflammation, are not yet established with sufficient precision to enable us to determine with perfect confidence under which of these heads some doubtful forms of disease should be classed. Many diseases, loosely attributed to irritation alone, are often characterized by symptoms which a more accurate diagnosis would enable us at once to ascribe to actual inflammation. In the description of the irritable uterus as above quoted, we encounter several symptoms which are known to be constant accompaniments of inflammatory action. All the occasional causes of the disease, as enumerated by Dr. Gooch, as well as the greater number of its essential symptoms, would seem to lead to the supposition of a proximate state of parts, if not actually inflammatory, at least one of no inconsiderable vascular congestion; for in addition to a morbid state of the nerves of the affected organ, which is not disputed, there is also unquestionably a morbid over-destension of its blood-vessels during the presence of this disease: and this is, after all, the point of greatest importance practically to attend to, inasmuch as it bears immediately on the principal feature of the treatment to be adopted. The designation given by Dr. Gooch of 'the irritable uterus' to the distressing malady which he has here so admirably described, is therefore so far objectionable, as it leaves out of view one of its original, and perhaps its very principal constituent, viz. A PAINFUL OVER-PLENITUDE OF A PART AT LEAST OF THE INTERNAL ILIAC AND PUBIC SYSTEM OF BLOOD-VESSELS. Such a condition of the blood-vessels in question is more or less an obvious result of the occasional causes by which the disease is represented as being most frequently produced. It is promoted and exasperated by whatever exertions or other cuses which may be supposed calculated to increase the over-destension of the uterine vessels. The author recollects the case of a painful affection of the right foot, which was incurred by a gentleman some eighteen years ago by over-exertion in walking. At first the pain was considerable, and greatly interfered with the gentleman's pursuits; which required much personal activity. It was subject, like that of the irritable uterus, to occasional abatement, according to the degree of rest which could be afforded to the effected limb, and to more or less exacerbation, according to its exposure, which, indeed was unavoidable, to more or less of walking exercise. It was, however, at no time so severe as to render walking totally impracticable. For this reason the case was almost entirely neglected in the beginning. It consequently became a chronic affection, which, although it gradually abated of its original violence, has never altogether ceased to occasion inconvenience. Now the reader will easily recognise something of analogy between the occasional causes respectively of the irritable uterus and of the lamed foot. If the painful effects be not of a nature to be identified with a state of inflammation in the one case, it would of course be quite proper to dispute its existence in the other. On the other hand, an over-extension of tissue in the one case might be expected to produce a similar result as to proximate effect to what is known to take place in the other. In the foot case a state of exhausted power was followed successively by an over-extension of fibres and a slow subacute inflammation of the injured tissues. Of the fact of the latter result, the author has most abundant reason to be quite certain. But why admit such results in the one case, and deny or totally overlook them in the other? Of the foot case the proper treatment undoubtedly would have been the application of a suitable number of leeches to the part, and the immersion of it for an hour or two afterwards in hot water, or the assiduous application for an equal length of time of hot fomentations to the surface, followed by a repetition of the same practice on the next, or on an early day, subsequently; giving also to the limb the benefit of two or three weeks' most perfect rest. But it may be very well asked, whether the idea of exclusive irritation could be supposed so directly to lead to the proper practice in such a case, as that of inflammation, or of that even of congestion of the vessels of the part consequent upon the application of the previous injury. The author thinks not. For the same reason he therefore thinks that the new designation of Dr. Gooch, as applied to the morbid condition of the uterus, which in many respects he most faithfully described, may have the effect of leading practitioners to an inert and procrastinating practice.—About two years ago a case occurred within the cognizance of the author, very well suited to illustrate the tendency of a name to impose upon a weak mind, in an affair precisely of the kind, or rather in the instance of the very disease which we are now describing. Mrs. S. of B. Crescent, a very delicate lady, of about thirty years of age, and the mother of a numerous young family, had been the subject of much uterine irritation for about eight months, for the relief of which nothing very efficient had been done by her ordinary medical attendant. The husband, without giving any intimation to that gentleman of his intention, requested the present reporter of the case to pay his lady a professional visit, and to favor him with his opinion of the nature of her malady, and of its probable issue. The neck of the uterus was found exceedingly painful and considerably swollen; but without structural disorganization. The patient was greatly attenuated, and very pale and spiritless. The case was reported as one of no urgent danger, but nevertheless one involving some ultimate risk, if the present symptoms, which were represented as those of a peculiar variety of inflammation could not be subdued. On being requested to see the patient again, he suggested the propriety of his being met by the family medical attendant. But that person was unappeasably offended at the husband for requesting another opinion without previously consulting him and without his consent, and declined all further attendance on the case. In a short time, however, afterwards, upon learning the author's opinion, he took great pains to represent it as being totally unfounded; adding, that if it should be acted upon, the practice would soon prove fatal to the unhappy patient. The neck of the uterus, it has been already stated, was considerably swollen. With the aid of a speculum, it was seen to be also in a state of intense superficial inflammation. All its vaginal portion was of a vividly red color, similar to that of external genital surfaces when become the seat of a recent gonorrhœal affection. A quantity of viscid mucus was seen distilling from the uterine orifice. Little intimidated by the angry oracular prognostics of his predecessor, the author hesitated not to order four leeches to be forthwith applied to the orifice of the uterus. This duty was perfomed by the very intelligent midwife of the Maternity Charity, whose useful services in this respect he has already had occasion to notice. He was induced to limit the number of leeches to four, in consequence of observing how intensely the vaginal part of the organ was charged with blood. The quantity of blood obtained amounted to at least ten ounces, and the abstraction of it was almost immediately followed by the happiest results. After an attendance of about three months, during which the application of between four and six leeches were repeated four or five times, the author on retiring had the pleasure of leaving his fair patient in a state of much comparative comfort, of almost total freedom from the distressing pain of the uterus which had recently embittered her existence, and in other respects rapidly recovering her former health and strength. There was in this case very probably the irritability of the uterus, which had been represented by the family attendant as the patient's peculiar malady, but which had been in no degree mitigated by the soothing and strengthening medicines exhibited by him for its relief; but there was also most unquestionably much positive inflammation of the vaginal portion of that organ, the removal of which, by the depleting measure already described, made way for the eventual subduction also of the accompanying irritability. In the case of 'THE IRRITABLE UTERUS,' there is a period of recency and comparative acuteness of symptoms as certainly as there is in those of the irritable tumor of the breast, and of painful affections of knee and ankle-joints from over-extension of their ligaments; and there is little doubt but early and efficient vascular depletion would be quite as beneficial in all cases of the former, as they would probably prove in either of those of the latter. But would the hypothesis of the mere irritableness of the part in any one of these cases directly lead to such a practice? Again, the author thinks not. He accordingly finds local bleeding placed by Dr. Gooch under his second head of remedial measures; whereas the supposition of an over-fulness of the vascular system of the affected organ would naturally point to the relief of such a state AS A FIRST MEASURE. But if the disease be one of irritation and not of inflammation, nor of any condition of the parts allied to that of inflammation, why bleed at all? Because probably the utility of the practice had been fully ascertained by experience before the theory of the irritable uterus had presented itself to the mind of its talented propounder."

From the above observations it is evident that the author considers vascular depletion as forming the main feature of the treatment. But general blood-letting, he thinks, can rarely be necessary. The disease is local, and local abstraction of blood from the os uteri, he avers to be the best remedy. The complaint is usually concealed for a long time, and consequently the cure is rendered thereby tedious. The application of four leeches to the os uteri will generally secure the abstraction of eight or ten ounces of blood, and be succeeded by relief of the symptoms. The second most important measure is the horizontal position—and these two means, if early had recourse to, would soon reduce the disease; but when become chronic, then the recovery is tedious.

"There is, however, one point of practice, in reference to this form of the disease, to which the reader will do well to pay particular attention. The subjects of "the irritable uterus," are not always unsusceptible of impregnation. On the event of conception taking place during a period of remission of its most urgent symptoms, the medical attendant should then more than ever, and especially during the earlier months of gestation, insist upon the strictest conformity to his precepts in respect to the observance exclusively of the horizontal position. The action of gestation introduces a great change into the uterine system. During the last four months it places the uterus in a situation to be in a great measure secure from the attacks, if not altogether beyond the reach of some of the most influential occasional causes, of the disease. On the completion of the process of parturition, the patient may indeed be said, in reference to her former complaint, to have the opportunity of commencing a new life. If, during that period, she could be induced to keep her bed, in the most literal sense of that expression, for six weeks or two months, she would almost certainly secure herself against a relapse of her complaint subsequently to her confinement. In consequence of the prodigious development of parts interested in the business of gestation, nature is observed to exhibit a power of self-restoration and adjustment during the puerperal states, which at no other time nor under any other circumstances does she seem competent to exert. Hence, in cases of moderate prolapsion of the uterus incurred by forward conduct, during one confinement a perfect cure may frequently be obtained by the patient confining herself to her bed, and maintaining rigidly the horizontal position for at least five or six weeks subsequently to her next delivery."

Anodynes are necessary, and opium is the most efficient; but as it too often confines the bowels, and checks the biliary secretion, hemlock, hyosciamus, &c. combined with camphor, become necessary as substitutes. Battley's laudanum thrown into the rectum will often succeed, where it is found to disagree if taken into the stomach. As an aperient, Dr. Davis recommends sulph. magnes. in infus. rosar.—castor oil—electuary of senna—sulphur. In some cases of irritable uterus, accompanied by obesity, Dr. D. has seen good effects from mercury, as an alterative.—*Medico-Chirurgical Review.*

2. *Discharge of Worms from various parts of the Body.**—John Alexander, aged 10 years, was for nearly a year in a delicate state of health; although his appetite continued pretty good, he had much wasting of flesh and general debility. About eight months since a tumor arose over the epigastrium, which after being poulticed, for some days, burst and discharged, with about two ounces of pus, a white worm half an inch long. In a few days the abcess healed. Eight or ten days afterwards a second tumor arose about three inchess distant from the first, on the right side of the chest, which after some days also burst and gave exit to another worm. It is needless to particularize the different instances, suffice it to say, from the time of the first worm being discharged until I first saw him, which was an interval of two months, five worms had made their appearance. They were all similar to the first, and lived for a few hours after their discharge. When I saw him the integuments of the right cheek and eye were excessively swollen, and in the course of a few days another worm was discharged from the upper eyelid. I recommended different medicines for the space of six weeks, but the formation of abscesses on different parts of the trunk and extremities proceeded, and altogether about twenty worms were discharged, principally from the right side. At length two grains of calomel were given every night until the gums became affected, and convalescence shortly afterwards took place, I shall not pretend to say whether from mercurial influence, or from the produce of the original nidus having been exhausted. The boy has been for now nearly three months quite well, his health and strength being completely re-established. The worms appeared to be ascarides. None were, however, at any time observed to be discharged from the intestines, nor were the bowels irregularly affected.

I cannot account for their formation: whether the first deposition of eggs had been made by some means under the external skin, or whether a worm had perforated the intestine, and at length made its way to the surface. I incline to the latter opinion from the boy's previous ill-health. I could in a few instances trace a reddish line from one abscess to the other, this was, however, after the new abscess appeared; the patient himself felt no uneasiness in the part, nor had he any idea where the next abscess would form until it appeared externally.—*Ibid.*

3. *Mr. Robertson on Dry Cupping.*†—Mr. Robertson, an intelligent surgeon residing in High Holborn, has published some interesting cases of pain depending on various causes relieved by dry cupping. The cases in which he would recommend it are those in which the pain is dull though severe, deep-seated, chronic, not much increased by pressure, or has refused to yield to ordinary means. The way in which he generally employs the remedy is to throw a very minute bit of paper touched with ether or turpentine, lighted, into a large glass

* Extract of a letter from Mr. C. Neilson of Killala.

† Lancet, January 19th, 1833.

or tumbler, and press it down in the usual way. This is a very effectual and a very convenient method of obtaining the requisite exhaustion, and is attainable when the regular cupping apparatus is not. We will select two cases out of eight related by Mr. Robertson. They are the most interesting and perhaps the most satisfactory.

Case 1. *Spasmodic Pain in the Loins, post Coitum.*—"A stout young gentlmen, and in fine health, was seized shortly after connexion, with most violent deep-seated pain in the region of the *left* kidney; so severe that he was unable to walk, stand, or sit. Lying on his back on bed, or on a sofa, gave a little, and only a little, relief, and frequently did not relieve him at all. He had been often attacked similarly before, and traced it distinctly to connexion. The peculiar sensation in the part commenced immediately after coitus, and could be felt distinctly increasing more and more till it ended in a paroxysm. Sometimes the sensation went off altogether, particularly if he carefully kept the recumbent position; but when it did not, four or five hours would intervene before the paroxysm came to its height. In one instance he was attacked severely while walking home, and had very nearly fallen down in the street. In another he was awoke in the night. Twenty or thirty leeches relieved it the first time. In about two months it came on again, when bleeding at the arm and 70 leeches over the seat of pain, gave him only moderate ease.

After some month's interval it returned a third time, and leeching, even to a very great extent, seemed to have altogether lost its power. A blister and some internal medicines were prescribed by a physician with little relief, when I ordered 20 leeches to the anus; this, and the recumbent position for two days subdued it.

A fourth time he felt it coming on. This was at midnight, (connexion having taken place some four hours previously,) and when I saw him, the pain in the left lumber region seemed frightful, deep-seated, and of that peculiar nature, that he could bear almost any pressure on the part without shrinking. He lay in bed writhing like a serpent.

I felt loath to bleed him to such an extent as had been requisite formerly to subdue it. Leeches were not at this time conveniently to be had, and I determined to try the effect of dry cupping A very large tumbler was put over the part, kept on for a minute or two, till it seemed to gall him; taken off, and replaced three or four times. The effect delighted and almost astonished me. One minute after it went on, the most perfect relief was felt; the pain was entirely gone; so afraid was he of its return, and so keen to have the glass on, that he insisted on having it on and on, till the edges of the tumbler had almost cut into the muscles! This he declared he cared not for. It was a trivial thing compared to the dreadful and insufferable pain in his side. I knew him for years afterwards, and though the cause was continued as before, he never had any return."

Mr. Robertson susposes, reasonably enough, that the pain in this instance depended on spasm of one of the lumbar muscels.

Case 2. *Pain in the Left Umbilical Region.*—"A very interesting young lady, the wife of a friend of my own, left —— for Edinburgh, immediately after the marriage ceremony was performed; and when about half way, was seized while in the carriage, with most violent pain in the left umbilical region. Her husband managed to get her by easy journeys to Edinburgh, where she remained about three weeks, and was bled, blistered, purged, and put through all the ramifications of the strictest antiphlogistic system.

At length she was obliged to be brought home by short stages, a distance of 40 or 50 miles; and was ultimately relieved by turpentine enemata, which brought away some discolored hardened feces. Her menstrual periods had for years been attended with extreme pain. During the two years which followed marriage, she was said to have had two miscarriages. During the third she miscarried again, and was getting round, when I was suddenly sent for, on account of an alarming pain *in the very spot which had formerly been so productive of suffering*. She could not account for it; she had been lying quietly in bed, and had been eating and drinking nothing to produce it. The lochial discharge went on as usual, and her bowels were natural, as she had, ever since her former attack, used Maw's instrument. Leeching was proposed, but to this I objected, on account of the loss of blood she had sustained so lately, and from the effects of which she had not as yet recovered.

I determined at first to try dry cupping; she ascented, and as the things are always at hand, in five minutes she was so well as to be able to joke with her husband and me about 'the very troublesome wife' the former had. It never returned."—*Ib.*

4. *Use of Turpentine in Sciatic Neuralgias.*—M. Martinet has adduced a long catalogue of cases to show the superior efficacy of small doses of the oil of turpentine. To prevent its acrid effects on the stomach and bowels, he recommends that it be always blended with some corrective excipient, such as honey, gum-arabic, magnesia, yolk of egg, &c. and the dose ordered is a drachm or two in divided doses, daily. As a matter of course a correct diagnosis must have been previously made, in order that we may be satisfied that there is no inflammation or organic disease of the nerves; under such circumstances we cannot reasonably expect a cure from the turpentine alone. If the drug vomits or purges to excess, opium should be added. In 40 cases of acute neuralgia, 34 were cured, 5 relieved, and 1 was not benefitted. In 31 chronic cases, 24 were cured, 3 were relieved, and 4 experienced no advantage: 33 of the cases treated with the turpentine had resisted other remedies previously employed. The period generally required for the cure was from 5 to 12 days; in a few cases the medicine must be continued longer. Out of 58 cases 48 were sciatic, 3 crural, 4 brachial, and 3 facial neuralgias.—*Bullet. de Therap. from Medico-Chirurgical Review.*

5. *Lisfranc's Treatment of Amaurosis.*—First of all, we should ascertain whether there are any symptoms of inflammatory fulness and activity in the eye or head;—as a matter of course, such cases require depletion; when, however, we have reason to believe that the disease is one rather of debility, Lisfranc strongly advises us to direct our attention in an especial manner, to stimulate the frontal and other branches of the fifth pair of nevers by means of repeated blistering over the eyebrows and temples. Should this fail, we must endeavor to excite the torpid organ by acting immediately on the ciliary nerves, any irritation of which is speedily propagated to the ophthalmic ganglion and the origin of the trigeminus. This is most effectually done by the application of stimulants to the cornea; and of these stimulants the nitrate of silver in substance is the best. The inferior segment of the cornea is to be lightly touched, till we perceive a whitish cloud;—the eye is then to be immediately washed with water. Considerable pain is felt; the whole apparatus of the eye is put into a state of so increased activity, that on the morrow a stranger might suppose that our patient labored under acute ophthalmia. This treatment induces sometimes vomiting; and as it always occasions temporary contraction of the pupil, it must not be employed when there is a tendency to this evil. The operation requires to be repeated several times.—*Journ. Complem.—Ib.*

6. *On Hydatids, and their Conversion into Tubercles.*—M. Kuhn has lately read before the French Academy a memoir on acephalocysts, and the manner in which these parasitical productions give rise to tubercles. He holds the opinion of Laennec, Bremser, and others, that they are to be considered as truly of an animal nature; and draws a distinction between those found in the human body, from what are often seen in sheep and other lower animals; the former, says he, are always propagated by internal buds, or growths which are thrown off from the inner surface of the original hydatid, and may be, therefore, denominated "endogenous;" they may be compared to a nest of boxes, one within the other, whereas the latter produce only on their outer surface, and are, therefore, "exogenous." It sometimes alone, sometimes simultaneously was after a very careful examination of the lungs of oxen, which had died of a species of phthisis called "pommeliere," that M. Kuhn was led to the belief of the degeneration or conversion of hydatids into tubercles. The hydatids, by their irritation, causes cysts to be formed around them; these cysts become stronger, fibrous, or even cartilaginous; meanwhile, the acephalocysts enlarge by serous imbibition, and multiply by buds from their inner surface; these again, in course of time, give rise to others, the whole nest being contained in one bag. From the inside of this bag is secreted a yellowish viscid matter, which becomes thicker and thicker; M. Kuhn regards it as the primitive tuberculous deposit; it gradually solidifies, and, with a simultaneous shrinking of the cyst, tends to squeeze and kill the enclosed animals, thus giving rise to a nucleus of tubercles. Sometimes the tubercles are not entirely filled up, but are hollow, and we observe only the shell or dried husk of the acephalocyst; we may even separate the thin layer of the animal from the debris within, by immersing some of the tubercles in water. M. Kuhn has enriched his memoir with beautiful illustrative drawings; they throw much light on the etiology of the tubercles which are found in the lungs and liver of ruminating animals. The co-existence of hydatids and tubercles, in the same organs, is a fact at once curious and most interesting. The subject is one of much importance, and deserves future examination.—*Revue Medicale.—Ib.*

7. *Chloride of Lime as a Lotion, against the Itch.*—Professor Fantonetti gives the result of nine trials. In seven of these cases the disease was cured in from six to eight days; in the 8th case the psorous eruption was removed, but was followed by an eczema, which gave way under the use of tepid baths; and he left the hospital well; the itch however reappeared, and then yielded, only to sulphureous fumigations. In the 9th, the psora was cured—re-appeared—was cured again—returned a third time—and was finally subdued by the sulphureous fumigations. The chloride of lime lotion is made, by adding 1½ or 2 ounces of the chloride to a pint of water; it ought to be rubbed three or four times a day on the affected parts. A simple tepid bath is to be used every third day, in order to soften the skin, and clear it of any calcareous crusts. If the lotion is too irritating, its strength must be reduced. The disease is generally cured effectually in eight days.—*Annali Universali.—Ib.*

8. *Dupuytren's Mode of Treating Prolapsus of the Rectum.*—This eminent surgeon has cured a vast number of patients laboring under this disease by means of an operation which is both simple and speedily effectual. He seizes the folds around the anus with forceps whose blades are large and flat, and excises them with strong sharp scissors; this excision must be carrid deep enough to remove not only the integuments embraced by the forceps, but a portion of the rectum, when the relaxation of its mucous coat is very considerable; generally the depth of the wound need not exceed a few lines, but in other cases it must be at least an inch. Dupuytren usually cut off in this manner four folds of the margin of the anus, one in front, one behind, and then one on each side; if the disease be not of great extent, the removal of one or two of the folds may be sufficient. This operation is seldom followed by any considerable hæmorrhage. It is necessary that the patient be so treated, that he has no occasion to have the bowels relieved for eight or nine days, in order that the wounds may not be disturbed. The cure is generally complete in a fortnight. Dupuytren has met with no case of failure hitherto.—*Journal Complementaire.—Ib.*

9. *Discovery of a New Principal in the Serum of the Human Blood.*—M. Felix Boudet, in a memoir presented to the Institute, has investigated anew the composition of the serum of the human blood. His researches prove, that when evaported to dryness, exhausted by boiling water, and dried anew, it yields to boiling alcohol the following substances; 1*st*, a peculiar immediate principle, called by the author

*seroline; 2d, cholesterine; 3d,* a *soap*, soluble in water, most probably formed by the margalate and oleate of soda; and *4th*, the *fatty matter of the brain.*

*Seroline* in deposited, on cooling, from the boiling alcohol by which the serum has been exhausted. The liquor filtered, when cold and evaporated, leaves a residue of a resinous consistence (*de consistence terebinthineuse.*) On treating this with clod alcohol, at 36° (*specific gravity* 0.8252,) the fatty matter of the brain is separated, and a substance is dissolved, which is considered by M. Lacanu to be an oil, but proved by M. Boudet to be composed of several distinct principles. In fact, the liquor left to itself, deposits crystals of cholesterine, and retains the soap mentioned above, together with a little of the fatty substance of the brain.

*Seroline* is white, of a slight pearly-color, in the form of filaments, which, viewed by the microscope, present the appearance of globules, or globular swellings. It melts into a colorless oil at the temperature of -|-36° (97.8. *Fahr.*) It has no action on colored re-agents, but like cholesterine, becomes red on the contact of concentrated sulphuric acid. Sulphuric ether dissolves it easily; alcohol on the contrary, at 36° (*sp. gr.* 0.8252,) dissolves only a trace of it at the temperature of ebullition, but has no effect on it when employed cold. Treated hot for six hours with *aqua potassæ*, it seemed not to experience any modification, and hydrochloric acid produced to the least disturbance in the alkeline liquor. Neither acetic or hydrochloric acid produced any apparent alteration on it, whether hot or cold. When heated a long time with nitrih acid, it was not dissolved, but became soluble in *aqua potassæ*, to which it communicated a brown color. Distilled by the lamp in a small glass tube, it diffused a very characteristic odour, furnished some alkaline vapors, a trifling residue resembling charcoal, and appeared to be in part volatilized. The small quantity of *seroline* obtained, did not permit M. Boudet to submit this substance to a greater number of tests, but he conceives that enough has been done to prove it to be a new principle.—*Ed. Med. and Surg. Journ. from Journ. de Pharmacie, June,* 1833. *from American Journal.*

10. *Pathology and Treatment of Gastritis.*—Extracts from a clinical lecture by Dr. William Stokes.—Numerous and important advances have been made in modern times, in the knowledge and treatment of gastric affections, and an enormous mass of facts have been accumulated of the deepest interest to practical medicine. It is, however, an unfortunate circumstance, and calculated to excite much surprise, that a knowelge of the various forms of this inflammation is not sufficiently spread among British practitioners. There is still a great deal of ignorance and misconception on this subject; many persons are still accustomed to take a limited and superficial view of it, and a thorough acquaintance with the various modifications of gastric disease is at the present day any thing but general. At one time we hear it called disease of the liver, at another time dyspepsia, sometimes it is termed constipation, and sometimes derangement of the digestive organs. It is true, that in such cases we find constipation, dyspepsia, and derangement of the digestive organs, yet these terms, as they are commonly employed to designate the disease, are at once both useless and improper, because they convey no correct or pathological idea. We owe, I think, to Broussais a great deal of our knowledge of gastric and enteric inflammation; it was his researches that gave the first clear and luminous view of a class of diseases previously obscure and little understood. He failed, however, in procuring the general assent of the profession to the propositions he advanced, and one of the principal reasons of this failure, and of the partial diffusion of the knowledge of gastritis in this country is, that although he brought forward a great number of valuable facts, he also promulgated a theory which has not been cleary or successfully proved. This theory has been rejected, and with the theory British practitioners rejected his facts. * * *

Gentlemen, we have had no acute cases of gastric inflammation of late in our hospital wards, but there are two cases of chronic affection of the stomach, on which I purpose to offer a few remarks. The first is the case of a man in the chronic medical ward. This man is from the country, and is at present laboring under an affection of the stomach, exceedingly common among the Irish peasantry. I have seen a great deal of it in the course of my own practice, and any person residing in the country, if he happens to be a medical practitioner, must have been repeatedly called on to treat this form of disease. At least persons, who labor under it, are chiefly of that class who live a good deal on potatoes, and I am inclined to think this exclusive diet must have some share in its production. Most of those, who come into hospital with this complaint, are from the country, particularly persons whose circumstances have been impoverished, and who consequently have been compelled to change their former nutritious and better food for a potato diet. In such cases, we generally find those persons able to date the commencement of their illness from a poriod (immediately) subsequent to this change, and it is therefore not improbable that the change of diet has some influence in the production of this peculiar gastric affection.

The patient, who is the subject of the case before us, is somewhat reduced in flesh and of a sallow complexion. He has complained of pain in the region of the stomach, extending to the back, right hypochondrium, and shoulder. He has had tenderness over the epigastrium, loss of appetite, pain and sense of distention, increased after eating; vomiting of yellow matter occurs two or three hours after taking food, succeeded by thirst. His pulse is soft and slow; tongue clear; bowels open. His illness, (and this, I think, is a point worthy of remark,) commenced four years ago with pain in the stomach, increased by eating and relieved by vomiting; and some time after this the vomiting began to be succeeded by thrist. The vomiting generally came on in an hour or two after taking his meals, and he threw up a quantity of yellow slime. Another important point is connected with the treatment, he has undergone for this disease. He has been relieved by antiphlogistic treatment, locally employed; we treated him since his admission by leeching, blisters, and cupping over the stomach, and latterly he has been using narcotics. Ninety-nine persons in a hundred would be inclined to call this a case of dyspepsia, and so it is so far as loss of appetite and derangement of the stomach are concerned, but the worst of the thing is that they would look at it only in a single point of view, and treat it as mere nervous disease of the stomach. Well, with respect to this man's case, it is either a chronic gastritis, or it is a nervous affection, and mere dyspepsia. Before we proceed further, let us see what is the precise meaning of these terms. Dyspepsia is a name given to that condition of the stomach, in which, without any sensible alteration of structure or circulation, the stomach does [illegible] perform its functions in a regular and proper manner, but there [illegible] no organic lesion; and if a man, laboring under simple dyspepsia, were to die, we could not detect any change in his stomach, so far as circulation and structure are concerned. Chronic gastritis is a lesion of the stomach, with a change of its circulation and a thickening of its lining membrane, or, in orther words, with signs of actual disease in the stomach. Now, in my opinion, there is a great probability that this affection, which is so frequently observed among the peasantry of this country, is a chronic gastritis. It may, I grant, commence by dyspepsia, but, in its advanced stage, and it is only in this stage that such cases come under the care of medical men, it is most commonly a chronic gastritis. We have heard, it is true, no post mortem examinations of this disease, and can, therefore, only reason on probabilities, but if we look to that form of treatment which has been found most successful in affording relief, we find it to be that which is calculated to remove irritation and vascular excitement. Besides, the antidyspeptic treatment has failed, after extensive and repeated trials. In fever this gastritis is of very common occurrence; and here also it is most advantageously met by local antiphlogistic means. But there is another circumstance which you should always keep in mind: by the time you get persons laboring under this gastric affection to come and place themselves under your care, they have been ill for months, and pephaps years. The disease is certainly at this time very chronic; and you are aware that it is a general pathological law, of the truth of which we have the most ample proof, that where an organ has been long subject to functional derangement, there is a strong probability that more or less of organic change has also taken place. We seldom see the brain, or the lungs, or the kidneys deranged in function for many years without more or less of structural alteration; and we may conclude that any viscus, in which functional disorder has existed for a considerable period, will ultimately experience organic change. If, then, we connect with these facts the failure of the antidyspeptic plan and other circumstances, we are led to infer that these and similar affections are cases of chronic gastritis. I do not say it is so exactly, but I think the collateral proofs are very strong in favor of its existence.

Well, what have we done in the present instance?—We have endeavoured to regulate the man's diet; we have cupped and leeched the epigastrium, and afterwards employed counter-irritation. In all cases of a similar nature our treatment has been nearly the same; in most it has been followed by permanent relief; but where this did not occur, and the patient complained afterwards, we have had recourse to narcotics. This man has been laterally taking, with the most signal benefit, the eighth part of a grain of acetate of morphia twice a day. You will see in Dr. Bardsley's Hospital Facts and Observations that the acetate of morphia has been employed with singularly good effects in the treatment of gastric affections; and where its use has been preceded by leeching I have a full conviction of its value, as well as that of various other narcotics.

There is, gentlemen, another case,—that of the patient Denham, who has been complaining of pain and tenderness in the epigastrium, with loss of appetite, and intolerable thirst. His face and extremities are œdematous, urine not albuminous, bowels confined. His tongue is red, and thickly coated with fur; his illness commenced two months since. I looked on this as a case of chronic gastritis; for, you observe, he had all the symptoms, pain, tenderness of the epigastrium, red tongue, impaired appetite, and an insatiable desire of cold drinks. We treated him by leeching and blistering the epigastrium; we gave no purgative by the mouth, but obviated the costiveness by enemata. By this treatment much good has been effected. Since the leeching and blistering, his thirst, which was so excessive that I thought at one time he had diabetes, has completely declined; his tongue is much improved, and he no longer complains of any gastric pain. His appetite, however, continues bad; and it will remain to be seen by the progress of the case whether this depends on want of tone in the stomach or actual disease.

In submitting these cases to your notice, it may not, perhaps, be irrelevant to make some observations on the pathology of gastritis in general. Many circumstances tend to prove that chronic gastritis is a very common disease. Although not admitting of direct proof, I think it is also true, that where dyspepsia has lasted for a long time, there is more or less of gastric inflammation. Nothing is more common than dyspepsia; and hence, in all probability, chronic gastritis is common also. We are not, however, to go the whole length with Broussais, and give the name of chronic gastritis to every case of dyspepsia which comes before us. Broussais is too much of a solidist,—he refers too much to the mere visible alteration of organs, and his idea is, that every case of dyspepsia is a case of gastritis; that there is scarcely such a thing as mere nervous dyspepsia; that all instances of this kind are only various forms of gastric inflammation, and to be treated as such. Here it is evident that theory has led him astray; for that this notion is incorrect has been proved by the circumstance that several cases of dyspepia have been relieved by treatment not calculated to remove inflammation. We every day see cases of dyspepsia deriving the most decided benefit from the use of stimulants, wine, and a generous diet; and where this occurs, who is there that would venture to call them chronic gastritis? But although we do not go the whole length with Broussais, and justly reject the speculative part of his doctrines, still we owe a great deal to his industry and research: he has brought forward a multitude of valuable facts which were formerly but little appreciated or understood; and when you are called on to treat a case of dyspepsia, I think you should take the greatest pains to ascertain whether it be chronic gastritis or not.

The next thing I have to remark is, that it is extremely difficult to make out the diagnosis of chronic gastritis; we do not know one symptom which would enable us to draw a line of distinction between dyspepsia and chronic gastritis. You will read in books a minute detail of the symptoms by which they are separately characterized, and will think yourself capable of making a diagnosis; but when you come to practice, even in an hospital, you will find the affair involved in very great perplexity. Another thing is, you will have all the symptoms and causes equally prominent in the early stage of both. I have often stated, that if I were examined on this subject, and required to give a diagnosis between gastritis and dyspepsia, I could not tell the diagnosis. The truth is, their symptoms are identical. In chronic gastritis there is no fever, and the pain, flatulence, distention, acidity, loss of appetite, &c. are the same in both. I feel convinced that chronic gastritis is very often confounded with dyspepsia by British practitioners. It is treated as disease of the liver by blue pill and black draught; it is treated as dyspepsia by tonics and stimulants; it is treated as constipation by drastic purgatives. Constipation arises from a variety of causes, frequently from inflammation of the upper part of the tube. Now, observe the result of mistaking chronic gastritis for any of these three complaints. If it be taken for dyspepsia, it must certainly be increased by the tonics and stimulants which form the great bulk of antidyspeptic remedies. Run over the whole class of antidyspeptic remedies, and you will find them to consist chiefly of powerful stimulants. If it be treated as disease of the liver, of course blue pill is given, but what is the consequence? The liver is stimulated, and there are copious bilious discharges; but the true cause of the disease, the gastritis, is wholly neglected, and, by neglect, becomes certainly worse. It will be also neglected, and even much exacerbated, if taken for constipation and treated as such. How are you to make the distinction and steer clear of error where your course is obstucted by so many difficulties? Recollect the rules which I have before given on this subject. If the disease is chronic, the probability is, there is more or less gastritis in it, and the more chronic it is the stronger is that probability. In the next place, when patients apply to you for advice they are generally a long time ill, and have gone through several courses of antidyspeptic remedies. Now, if you happen to get a patient who has been treated for months, or even years, with blue pill, bitter tonics, and stimulants, and find that he is rather worse than better, you have two data to go upon; your diagnosis will therefore be more likely to be formed safely and accurately, and your treatment successful.

You will ask me, perhaps, would I never employ tonics in the early treatment of dyspepsia? To this I will answer—never, in case it should be combined with gastritis. Here, however, I must remark, that Broussais has gone too far in restricting such cases to a pure antiphlogistic treatment throughout, for I believe there is a period when such treatment will do no good. When we have completely removed all irritation by the former plan, I think we may then have recourse to tonics with decided benefit. When we consider the curative action of tonics, stimulants, and bitter medicines, in the treatment of the majority of diseases where they are employed, we find that they are most efficacious and successful when preceded by a judicious antiphlogistic treatment. We shall see more of this as we proceed.

There is another case which I wish to notice: it has been, I believe, one of an acute character; I allude to that of the man in the Fever Ward. This person, after committing an excess in drinking, got sickness of stomach and vomiting. In your investigations of any case which comes before you, it is of importance, towards finding a cerrect diagnosis, to hold these two things in view—the exciting cause, and the first symptom of disease. Here you have, in the first place, excitement of the stomach from the use of spirits, and aftewards irritation, manifested by the vomiting. This was followed by loss of appetite, constipation, pain in the lower part of the left hypochondrium, foul tongue red at the tip, symptoms which indicate irritation of the mucous membrane of the stomach and intestines. When he was admitted into the hospital, however, what he chiefly complained of, and what were certainly the most prominent symptoms, were tightness across the chest, great difficulty of breathing, and harassing cough. His cough was indeed very severe, his sputa slightly tinged with blood, his breathing very much accelerated, and, to a superficial observer, he would appear to labor under chest disease. But, remark, we found not that he had been complaining of these symptoms for about three weeks, and consequently, if they had been pulmonary symptoms, they must have proceeded to a very alarming extent in that time. Mr. Lees examined him by percussion and with the stethescope, but could not detect any disease of the lungs, and he was examined by myself on the next day with the same result. Moreover, the patient had been previously treated for pulmonary disease without success. We were, therefore, led to conclude that there was no original disease of the lung, but only sympathetic irritation, depending on gastritis. We took small quantities of blood from his arm, leeched the epigastrium, kept his bowels open by enemata, and under this treatment we saw all his symptoms disappear, as it were, by magic. This is a remarkable case, giving us an illustration of the manner in which gastritis may simulate other diseases, and exhibiting the importance of attentending to the exciting cause and first symptoms of a disease in order to arrive at a correct diagnosis. Here you see it putting on the semblance of pneumonia, in other cases it assumes the guise of encephalities. It was remarkable, that the bleeding and leeching increased rather than diminished this man's strength, for after their employment his prostration nearly disappeared. You will see a great many of these cases in the course of practice, where the primary mischief is masked by a train of prominent sympathetic phenomena, and in which your diagnosis is to be founded on the following circumstances. These persons labor under a kind of fever; there is generally severe harrassing cough, and respiration is considerably accelerated; their symptoms have been of several days' duration, and when you examine them with the stethescope, you find that the pulmonary symptoms are not accompanied by corresponding organic lesions, and have not advanced in proportion to their duration. You either find no disease at all in the lungs, or a bronchitis too slight and trivial to account for such alarming symptoms. You next examine the larynx, and finding there no evidence of morbid change, you look for the cause of the cough in the digestive tube, and most commonly trace its existence to a concealed gastritis. If you meet with a case in which violent cough, labored respiration, and other symptoms of pulmonary disease have existed for a considerable time, without any signs of disease of the lung sufficient to account for them, you may often sit it down as a stomach affection, and direct your treatment acccordingly. Generally speaking, this simulated phenomena is most commonly met with in children, but instances of it in adults are by no means rare. Another thing is, if you happen to have tried pectoral medicines, and found them to fail, your diagnosis will be more certain. You remarked the case of a man who was here some time back, laboring under what may be called a tussis firma; well, this was dependent on gastritis. We treated him with leeches to the epigastrium and iced water with most signal benefit. He committed some excess in eating, and had a return of his complaint; he was treated again in the same way, and recovered. I remember having attended a lady some years ago, who complained of some feverishness, with very severe and harassing cough. Not being aware of the nature of the disease, I treated it as a case of fever, with irritation of the bronchi. The fever declined, but the cough continued without amendment; I was much embarrassed by its obstinacy, when one day, happening to be in attendance, the lady remarked that she had been under Dr. Cheyne's care for a similar complaint, and drived much benefit from leeches to the epigastrium. On this hint I acted; the leeches were applied immediately, and my patient's cough entirely disappeared. You will observe this is the point to which I would direct your attention; consider that the diagnosis depends on the persistence of pectoral symptoms; consider that if it were disease of the lungs, it would, in the course of two or three days, produce lesions capable of being easily perceived. But if this be the case, and you look in vain for any organic change to account for such excessive cough, you will seek for its cause elsewhere, and refer it to sympathetic irritation, produced by disease of some other organ, and this is most commonly the stomach. In connexion with this, I have to notice a very interesting fact in the pathology of gastritis. In such cases as the above, you will generally find but little direct evidence of gastric irritation. The patient has no vomiting, he complains of very little pain, and the epigastric tenderness is very slight. Here is the law by which such affections are regulated. In those cases in which the sympathetic irritation is most strongly marked, the usual or local symptoms of the disease are least apparent. We see cases of this kind apparently consisting of chest disease, and sometimes even assuming the appearance of cerebral disease, or tetanic symptoms, while the true signs are completely masked. You will find in Andral's work, a remarkable case of this kind, in which the ordinary symptoms of fever, vomiting, pain, epigastric tenderness, continued for a few days, when tetanic symptoms set in, and immediately those which were indicative of gastric irritation disappeared. But we are not to be deceived by the supervention of sympathetic irritation manifesting itself in other organs, nor are we to suppose that the gastric affection has subsided because we have an imposing train of symptoms existing in other parts. As long as the irritation continues, no matter in what organ it appears, we have strong evidence that the disease, though lurking, is still unsubdued. It is of importance to bear this in mind when you come to treat cases of sympathetic irritation depending on gastritis. If you treat them as pulmonary disease, as any superficial observer, or any person unacquainted with the use of the stethescope would be liable to do, your mistake will be, indeed, a very serious one. In the first place, the gastritis will inevitably be increased by being neglected. In the next place, though the internal remedies which are ordinarily employed for the removal of pulmonary affections, as tartarised antimony, squill, and other similar means, obviously produce the worst effects in gastric disease, and must tend materially to its exacerbation, so that there are in such instances two sources of exasperation, one arising from neglect, the other from the employment of therapeutic means which are totally contra-indicated by the nature of the disease. Remember, therefore, that where there are violent symptoms of disease of the lungs, and where these have gone on for several days without any porportionate lesion of these organs, that you may look for their cause and origin in a concealed gastritis. Recollect also, that in such cases the gastritis may be nearly latent, and want most of those symptoms by which it is generally characterized.—*Lond. Med. and Surg. Journ. May 25th,* 1833, *from Am, Journal.*

11. *Inflation of the Bowels.*—Mr. Blacklock, in a communication in our cotemporary, the *Glasgow Medical Journal*, for May, 1831, states that about thirteen years previously he attended a child whose bowels could not be moved by any of the agents usually employed in cases of obstruction, although there were no symptoms of inflammation till towards the close of the scene. Having obtained leave to examine the body, nothing could be discovered to account for death, but a very complete intussusception in the course of the ileum, and which immediately disappeared on inflating the bowels with the blow-pipe. It occurred then to Mr. B. that this might have happened had the bowels been inflated during life. Mr. B.'s son, about three years of age, was attacked with constipation, so that during nine days no evacuation could be procured from his bowels, notwithstanding the most persevering administration of purgatives, and the almost hourly use of enemata. At length Mr. B. had recourse to inflation, and the child immediately had a free evacuation. Mr. B. has since tried the remedy frequently, and often with the best result.—*Ib.*

12. *Iodine in the Treatment of Salivation.*—In a recent number of

*Hufeland's Bibliothek der Practischen Heilkunde*, there is a note strongly recommending iodine in cases of severe salivation, which is represented as removing the most violent inflammation of the salivary glands, and even healing ulcerations produced by mercury within a few days. The dose is two grains a day, increased to four. The following is the formula: ℞. Iodine, gr. v. solve in spt. vin. rect. ℨij.; adde aq. cinnam. ℥ijss.; syrup. simp. ℥ss. Dose, half a table-spoonful, and gradually increased.—*Med. Gaz.*—*Ib.*

13. *Inflammation of the Mucous Membrane of the Bowels.*—Extracted from the Clinical Lectures delivered at the Meath Hospital. By Dr. William Stokes.—This case would appear at first sight somewhat perplexing; but, by considering that this patient has had no irritability of stomach or vomiting, that during the course of his disease he has had thirst, but no desire for cold drinks, and that symptoms of irritation of the lower part of the bowels have been absent, you will be able to infer that it must be inflammation of the intermediate part of the digestive tube. This patient is, however, still in a precarious state, though he derived much benefit from the application of leeches to the right iliac region, and his head-ache rapidly subsided after their use; his tongue is still foul, and he continues very feverish. On Saturday he was very ill; he complained of ardent thirst, his respiration was fifty in a minute, but the stethescopic signs of disease were insufficient to account for such acceleration of breathing. Now, you all will recollect, that I have often told you that where there is fever and extremely hurried respiration, without any distinct evidence of disease of the lungs or windpipe, we should always look for the source of the disease in the digestive tube, and that this is most commonly found to reside in the stomach. In this man's case we could not, by the stethescope or percussion find any cause for the increased rapidity of respiration; but we observed that his belly was swollen and his thirst urgent. We applied the leeches again on yesterday with the most extraordinary benefit; the head-ache, tympanitis, and labored respiration were manifestly relieved. An objection might be raised to the efficacy of this mode of treatment, as the improvement took place on the fourteenth day, and it might be said that it was an improvement which depended on a crisis. To this I will answer, that I have seen so many cases of improvement after leeching without crisis, that it is unnecessary to take this into consideration, and that in the present instance there has been no crisis is obvious, as the patient is still in a bad condition. If all his symptoms began to decline on that day, then indeed the effect of crisis might be reasonably inferred, but his original affection still continues; and therefore it is fair to conclude that his improvement is attributable to the remedies employed. There is another point with respect to leeching in gastro-enteritic fever; *we have seen numerous instances of crisis brought on by the application of leeches to the abdomen.* This is a curious circumstance; but I have seen it occur in so many cases that I feel convinced it would not have come on if the leeches had not been applied. I have seen the application of leeches and the supervention of crisis in such close and constant connexion, that I look on them in the light of cause and effect. Can we explain this? If you look to those diseases which have a tendency to terminate by crisis, you will find that they consist of cases in which there is no great preponderance or excess of irritation in any particular organ. Of this simple typhus is one of the most remarkable examples. When there exists a decided point of irritation in any particular part, the tendency to terminate by crisis is much less. Thus we seldom observe a distinct crisis in cases of acute enteritis or hepatitis, or inflammation of the peritoneum. Whenever we bring on a crisis in any disease in which there is distinct irritation of some particular organ or organs, we generally accomplish our purpose by reducing the local inflammation, and placing the organs in such a state as to give nature fair play. This is a point I have not seen sufficiently dwelt on in any medical work, but it is one of great importance, and which I wish to impress upon your mind. I have seen the application of leeches to the abdomen so frequently followed by a crisis, that I consider it fair to connect these occurrences in the relation of cause and effect. In these cases of the secondary inflammation of fever, it would seem that the tendency of the general disease to terminate by crisis, is prevented by the intensity of a local inflammation, which by its sympathetic irritation keeps up a febrile action. Now if you modify or remove altogether this local affection, you, as it were, reduce the fever to the state of simplicity, and allow the tendency to a critical termination to operate.

A few more observations on this case are necessary. This patient exhibited one peculiar symptom, not generally described in these cases, a very evident pulsation of the abdominal aorta and the vessels which it sends to the viscera of that cavity. It appears that this is a circumstance of common occurrence, and that in most cases where there is acute local irritation the arteries going to the affected part take on an increased action independent of the heart's impulse. Thus, in cases of whitlow there is a manifest excitement observed in the arteries of the corresponding arm. The same thing I believe takes place in enteritis, and we may look on the increased pulsation of the aorta as arising from enteritic inflammation; when you lay your hand on the abdomen of a patient laboring under this disease, you often feel the vessels beating very strongly, *though neither the heart nor the pulse at the wrist is proportionally affected.* I do not say that we are to look on every case of pulsation of the abdominal arteries as the consequence of enteritis or fever, but where we find it occurring thus in fever, we are to conclude that it is indicative of disease in the bowels. We have constantly noticed this pulsation of the arteries of the abdômen to subside after the application of leeches; we have also seen it decline and increase in proportion to the existing disease; and I think we have many circumstances to prove and warrant us in concluding that it accompanies the disease of inflammation of the mucous membrane.

Another point which I look upon as somewhat new, is presented by this man's case. He had incessant thirst, but his desire was for warm drinks, and he refused cold. We may found a part of our diagnosis on this circumstance. In cases of acute gastric inflammation, patients are harassed by a burning thirst, there is an urgent desire and a constant demand for fluids, but these must be cold, the sufferer generally refuses all others. You will see in any work on toxicology, that in cases of poisoning by corrosive substances, which is only another form of acute gastritis, there is an insatiable desire for cold drinks. In the present instance we find our patient complaining of great thirst, but he prefers warm drinks, and never uses fluids in a cold state, a peculiarity from which I am led to infer that he has no gastritis, but that the inflammation is seated lower down in the digestive tube. When the inflammation is seated, say in the ileum, we have it in a part of the tube less sensible than the stomach. There is the desire for fluids, but not the demand for cold fluids. But when the stomach is the seat of disease, there is both the desire for fluids and relief from the direct refrigeration of the suffering organ. Another important subject for consideration may be noticed in this case. He has hand all through his illness more or less tympanitis, a circumstance to which I am anxious to call your attention, as connected with it is one of the worst errors in practice. From a dangerous habit of prescribing without taking the trouble of searching for causes, and from the universal leaning to specificism in medicine, many practitioners are in the habit of giving the spirits of turpentine when called to attend cases of this kind. Several cases have, indeed, been relieved by this plan of treatment, but I deny that tympanitis occurring in the acute stage of fever has ever been relieved by spirits of turpentine. We are to consider the tympanitis of acute gastro-enteritic fever as one of the consequences of inflammation, and its removal is to be effected only by removing the exciting cause. Can this be done by direct stimulation of an inflamed mucous surface with spirits of turpentine? Certainly not. If we give spirits of turpentine, the patient is purged, (frequently with great violence,) the tympanitis, too, disappears, but the next day we find a manifest increase of fever and thirst, the abdomen is more tender than before, and the tympanitis returns. You may give another dose, but if you do the fever assumes an alarming aspect, marked by the supervention of coma and delirium. Tympanitis we should always consider as one of the symptoms of acute inflammation, and never give turpentine in the commencement of the disease. In the advanced stage of the disease, where turpentine may be employed with benefit we find the tongue soft, and the abdomenal tenderness inconsiderable, and here the safest mode of emplying it is by injection.—*Lond. Med and Surg. Journal, July* 20*th* 1833, *from Am. Journal.*

14. *On the Efficacy of Dry-Cupping in Various Diseases.*—By R. J. Graves, M. D. Extract from a clinical lecture at the Meath Hospital.—I begin this day's lecture with some observations on dry-cupping, of which you have witnessed the trial in two or three cases at present in hospital. Most of you, I presume, are aware that dry-cupping has been lately recommended to the notice of the profession in a very ingenious and valuable paper published by Mr. Robertson of London; and, as it is a subject deserving of serious and interesting investigation, involving many considerations of practical importance, it will be necessary to notice it briefly, and offer some hints respecting its applicability to various forms of disease.

Dry-cupping is a remedy not by any means of modern invention; it was known to Hippocrates and Aretæus; and, in succeeding time, among the nations of the European contient and in the British dominions it was verygenerally employed, and formerly enjoyed the reputation of being a very fashionable remedy. Of late, it has fallen very much into disrepute; it is now very seldom employed, though some persons still use it, in hospitals and public institutions, where clinical experiments are conducted on an extensive scale. Mr. Robertson has attempted to revive this practice, and has proved that dry-cupping is a very valuable remedy, possessd of curative powers shared by no other therapeutic agent, and capable of being applied with advantage where the ordinary means are perilous or inadmissible.

Some time ago, Mr. King, of Stephen's Green, related to me the particulars of a case which exhibited, in a very remarkable manner, the benefit derived from dry-cupping. It was a case of hysterical vomiting, in a lady, for which every known remedy had been tried without any favorable result, and which was completely arrested by the application of dry-cupping to the stomach and margins of the ribs. This may appear strange to you, and you may be inclined to ask, how it is that a change in the condition of the integuments of the abdomen can affect the stomach? In reply to this I would ask, in inflammation of the stomach, whether acute or chronic, why is it that the application of leeches to the integuments relieves the gastric affection? In the latter, the result is equally strange as in the former instance; the circulation of the stomach is totally distinct from that of the integuments, and yet we have no remedy so efficient in relieving gastric inflammation as leeches, applied to the integuments of the epigastrium. Taking away blood from the surface produces a change in the circulation of the internal organs; detaining blood in the integuments in the neighborhood of any viscus, acts also on the internal circulation, and effects a corresponding change. Let us investigate this more minutely.

A cupping-glass is applied to some part of the body, and the air contained within it is exhausted by means of a syringe or by heat. In either case the integuments of the part are forced up into the glass by atmospheric pressure, so as to form a hillock, in which a considerable quantity of blood is detained, remaining in the capillaries of the part, and being, as it were, cut off from the general mass of the circulation. The experiments of Dr. Barry have proved the detention of blood in that portion of the integuments submitted to the action of the cupping-glass, and that the quantity so detained does not pass into the general circulation or partake in its changes. Now, if a given portion of skin has, in consequence of morbid action, an unusual quantity of blood thrown into it, and cupping-glasses are applied to the integuments in its vicinity, you draw off a great quantity of blood into the portion which you cup, and that part which presented an unusual quantity, in consequence of morbid engorgement, may be,

*pro tempore*, drained, and may, during the period of this application, make rapid progress towards health. The same observation holds good when you cup over an internal organ in a state of inflammation. You must be aware of the practice of tying arteries which go to tumors of various kinds, and that the application of the ligature has frequently proved successful in arresting the peculiar inflammatory process by which such morbid developments are accompanied. Now, cupping acts as a kind of temporary ligature on the vessels of the part to which the glass is applied, including even the capillaries; and it is in this way that it tends to prevent the absorption of poisions locally applied.

Having said so much about the application of cupping-glasses, their *modus operandi*, and their action as local applications, let us see how far the principle may be pushed, and also whether this mode may not be applicable to local affections alone, but also act on the general circulation in such a manner as to produce those effects which are commonly attained by different means. Dr. Arnott, in vol. i. p. 574, of his work on the "Elements of Physics," makes the following important observations on this subject:—"Reflection upon these circumstances led me to think that, in certain cases, the beneficial effects of blood-letting might be attainable by the simple means of extensive dry-cupping; that is to say, by diminishing the atmospherical pressure on a considerable part of the body, on the principle of the cupping-glass used very gently, and thus suddenly removing for a time, from about the heart, a quantity of blood, sufficient, by its absence, to produce faintness. The results of trial have been such as to give great interest to the inquiry, and the author's leisure will be devoted to the prosecution of it. An air-tight case of copper, or tin plate, being put upon a limb, and made air-tight by a leathern or suitable collar, tied at the same time round its mouth and the limb—on part of the air being then extracted by a suitable syringe, in an instant the vessels all over the limb become gently distended with blood; and, as the blood is suddenly taken from the centre of the body, faintness is produced, just as by bleeding from a vein. The excess of blood may be detained in the limb as long as desired, for the circulation is not impeded. To produce a powerful effect with a slight deminution of pressure, more than one limb must be operated on at the same time." From this it appears, that if you take the whole arm or leg or thigh of a man and place it under this machine, then exhaust it of air, and detain one or two pounds of blood in the integuments, the same quantity is abstracted from the heart and general circulation, and the effect produced is the same as if you had suddenly drawn blood from the system to this amount. The strongest man will faint if you cup both legs. I think this view of the subject opens new ground in the field of practical medicine. You are all well aware of the effects, the truly beneficial and admirable effects of blood-letting, and you know also, that these depend not so much on the quantity of blood lost as on the impression produced on the general system. If we have to deal with an extensive and violent inflammation, we do not abstract blood by a minute opening, we make a large orifice, or we open a vein in both arms at the same time, we place the patient in an erect posture and endeavor to produce deliquium. It sometimes happens that the patient faints from fear, or before any considerable quantity of blood has been lost, and this faintness, as Dr. Arnott remarks, answers as well as that which results from venesection. This I can also testify, for I have seen all the good effects of bleeding, produced by the terror with which the operation frequently inspires persons of delicate or nervous temperaments. Now, by the machinery before described, a machinery by no means complicated, you are able to produce with certainty, such a powerful effect on the general vascular system, as to obtain all the benefit derivable from general blood-letting. Dr. Arnott mentions another but more objectionable way of attaining the same purpose, and one which is inferior in efficacy to the mode detailed. If you apply a bandage pretty tightly over the upper part of a limb, suppose for instance round the thighs, so as to prevent the return of blood through the veins, and then put the legs into warm water, the quantity of blood detained in the lower extremities will be such as to make the patient faint. This mode may be useful on some occasions but it is inferior to dry-cupping, and can only be applied to the extremities. There is another and very important point relative to the employment of dry-cupping, which stamps additional value on it from its applicability to cases calculated to excite much solicitude and anxiety in the mind of every practitioner. You have often seen cases of inflammation, in which our sole hope of safety, or even life, depends on checking the inflammatory process, when we stand doubting or perplexed, balancing the possibly fatal effect of blood-letting on a sinking frame, with the slower but, perhaps, more certainly calculated close of an inflammation, which attacks some vital organ, and affects the very sources of existence. If, in such circumstances, we could produce results similar to those which accompany venesection, would it not be a very important desideratum? Now, the employment of dry-cupping holds out to us a fair prospect of attaining this end, of cutting short a menacing inflammation in that particular state of constitution where blood-letting is a perilous experiment, and regulating the errors of morbid action without having recourse to the customary shock of sanguineous depletion. I do not know any better or more valuable auxiliary in the practice of medicine than this, or one which is capable of greater extension and improvement. There is not a single practitioner who does not remember how often he has been forced to bleed when he knew that he was doing so at the risk of his patient's constitution and life; there is no one who has not, on such occasions, anxiously sought some other means of accomplishing the same purpose; and as this is promised by the employment of dry-cupping, I think this matter should become the subject of extensive clinical experiment, and that no time should be lost in proceding to investigate the true properties of a remedy, which is likely to open a new era in medical practice. Cupping-glasses might be made of convenient shapes, for applying them along the inside or outside of the thigh or arm, and might be so large that, with the aid of a syringe, the intended effect could be produced in a few minutes. With regard to their operation in cases of local disease, I think we cannot extend their use too far. There are many cases of hysterical nuralgia, sometimes affecting the side, sometimes the spine, and other parts, which hitherto we have treated by bleeding, leeches, stupes, liniments, and blisters. Fomentations and liniments some times succeed in removing this affection, so do leeches, but frequently both fail, and we are obliged to blister, which often produces great irritation, without being attended by any decided benefit. Here it is very probable, that we would derive great advantage from dry-cupping in the neighborhood of the affected part. There is one form of this disease to which it is peculiarly applicable. The most annoying thing, perhaps, about which a medical man is consulted, are the head-aches of young ladies. These are varied and numerous beyond conception, generally connected with some menstrual irregularity and derangement of the intestinal canal, and forming a class of disorders which would require a good monograph more than any other I know of. Many practitioners get into disgrace with ladies on this account, and, as a natural consequence, with the community in general. Bleeding here is of very little use, and gives only a temporary relief, or even in many cases aggravates the existing symptoms. The best plan of treatment is to regulate the menstrual secretion, and attend to the state of the bowels. But I will say no more on this subject, for I might lecture on it without end. As to the head-ache, if you leech they get worse afterwards, if you apply cold lotions the same result; the best thing you can do, in my opinion, is to apply dry-cupping-glasses to the back of the neck and between the shoulders.* Let us see what dry-cupping has done in those cases which have been treated with it in hospital. A man of the name of Ryan, who has been a long time in hospital, suffering from violent pains, produced partly by rheumatism and partly by neuralgia, complained of very severe attacks of pain in the lumbar region, lower part of the belly, and thighs, but particularly in the lumbar region, on one side of which the pain and tenderness was excessive. This man had been mercurialized and blistered, he had 100 leeches to the affected parts in eight different applications, he had been stuped repeatedly, he had all manner of liniments and internal remedies I could devise. He was certainly somewhat improved by this treatment, but not so much as I wished. Well, this man has received the most marked benefit and relief of his sufferings from dry-cupping over the seat of the disease.

Another man, named Eustace, who had sciatica, which was cured by acu-puncture and afterwards returned, experienced considerable advantage from this remedy. In the case of a woman above in the fever ward, laboring under bronchitis, we have observed an amelioration of the pectoral symptoms after the application of dry-cupping. It appears to me that cases of pain and tenderness are not the only ones to which dry-cupping is applicable, but that we may employ it

* Dr. Graves has expressed his opinions on this subject more fully in a paper which will appear in the forthcoming number of the Dublin Medical Journal.

also with hopes of success in congestion of internal organs. Cupping over the chest, I think would diminish if not cut short the paroxysms of spasmodic asthma, of tussis senilis, and of the acute suffocative catarrh. In bronchitis with emphysema, it would relieve the congestions of the lungs, and lessen the dyspnœa; and in the violent suffocating bronchitis of children soon after birth it, seems to be particularly valuable from its rapid effects. In the tremendous and fatal dyspnœa which accompanies this affection in children, bleeding and leeching are objectionable, from the danger attendant on them, and from their tedious operation, and are decidedly inferior to the prompt and efficacious agency of dry-cupping, which is free from any danger. You will be convinced that I do not overrate the value and advantages of dry-cupping, when you recollect the case of a man in the hospital who has empyema of the left side of the chest. In this case, which will be spoken of by my colleague, Dr. Stokes, the whole of the cavity of the left pleura is filled with matter; the heart has been pushed to the right side, and the man breaths only through his right lung. Now this man got bronchitis in his only sound lung, and you can easily perceive what danger he was in. It is obvious, that in such cases, from the long duration af the disease, the immense quantity of pus in the pleural sac, and the weakness of the patient's constitution, bleeding could not be employed without much hazard. We had recourse to small doses of tartar emetic and extensive dry-cupping over the chest. The result of this case, which I could not have treated so advantageously a fortnight ago, is very encouraging, for you have seen the relief this poor man obtained. It may seem to you that I am disposed to think too highly of a remedy, the properties of which are at present but little known; but, as I have stated to you before, its properties seem to be analogus to those of general and local bleeding, and it is of the utmost importance to investigate its effects thoroughly, and see if it is capable of the same application, and likely to be attended by similar results, or, if there be any differences in applicability, to know where the one and where the other may be employed with the greatest propriety and success.—*Lond. Med. and Surg. Journ. April 27th,* 1833, *from Am. Journal.*

15. *On the Obliteration of Veins as a Mode of Curing Varices.*—M. Davats, in a learned paper on the different modes of alleviating and curing varicose veins, objects, on various grounds, to all the plans hitherto proposed, and recommends obliteration of the affected vein by a novel procedure. This consists in irritating very slightly two opposite points of the internal surface of a vein, and, at the same time, in keeping those points in contact. A simple sewing needle of any description will answer for the operation, which is thus performed. First the vein is made to swell by means of ligature, is seized by the finger and thumb, and isolated by passing a needle transversely under it; then the anterior, and next the posterior, wall of the vein is pierced perpendicularly with the point of another needle, which is conducted higher up, to pierce a second time first the posterior and next the anterior wall. The last needle is retained in its place by means of a thread twisted like the figure 8. In five days the thread may be cut, and the needle will fall out of itself. The first needle may be left till it drops spontaneously. In a few hours after the expulsion of the needles, the little wounds will heal kindly.

M. Davats has made several experiments, which prove the safety of the operation, and the rapidity with which a radical cure may be thus performed, complete obliteration taking place by the fifteenth day. In one experiment the operation was performed on two parts of the same vein, leaving a portion between them filled with blood. The blood coagulated, and was absorbed; the vein became obliterated, and no accident occurred.

M. Davats maintans, that compression alone is insufficient to cause obliteration of a vein, and supports his opinion by several experiments, in which the most perfect compression failed to secure this result.

By the operation proposed, the vein is transformed into a cord, white, round, filiform, and analogous to ligamentous tissue. The obliteration extends above and below the point of lesion, as far as the first anastomosing veins, which become sufficiently dilated to give free course to the blood. Beyond the anastomoses the venous tissue assumes its normal state. No trace of inflammation is observed; no clot of blood found in any part; and the cellular tissue surrounding the portion of obliterated vein is found in the most healthy state.—*Edin. Med. and Surg. Journ. Oct.* 1833, *from American Journal.*

16. *On the Presence of Copper in Wheat and several other substances.*—M. Boutigny, by a very interesting and delicate process, has succeeded in discovering the presence of minute portions of copper in cider which had passed through a copper cock,—in cider, in the preparation of which no copper had been employed,—in the factitious waters of Seltzer and Vichy, prepared by several establishments of Paris,—in the water which had served to boil sorrel, artichokes, spinage, and succory, in a cauldron of yellow copper,—in common beef soup, prepared in a copper pot ill-tinned, and in a corresponding mass prepared in the same pot tinned anew,—in three samples of vinegar, and in six of brandy,—in claret, and in *vin de Chably*—and in treacle.

The process which he employed, consists in suspending, by means of a hair, the half of a fine needle in the midst of a liquid previously acidulated with sulphuric acid. The apparatus thus disposed, is placed under a bell-glass, and left by itself in an isolated apartment. As soon as the bar of iron is plunged in the liquid, M. Boutigny conceives that the action commences, though it is not till after six, eight, twelve, or even twenty-four hours, according to the state of the atmosphere, that it becomes apparent. Then the needle looses its metallic lustre. It is commonly at the head that the action commences, proceeding successively through the remainder, and terminating at the base. If the operation be carefully watched, at a certain period the superior half of the needle will be found tarnished, while the other half still retains its metallic lustre. Then will be perceived covering the needle some air bells, which become larger, and when they have acquired a certain volume, detach themselves, and burst at the surface of the liquid. In from one to three days this disengagement ceases at the surface of the bar of steel, to commence at the inferior part, a curious phenomena, which occurs only in liquids containing a notable quantity of copper, and which may be attributed to two causes, acting, perhaps, simultaneously, electricity, and the coppering of the needle. The latter opposes the disengagement of the gas by an action altogether mechanical, and the needle, in becoming polarized, does not permit the gas to be disengaged except by its negative pole. After this the head of the needle will be the positive pole, and the inferior extremity the negative. Admitting electricity to be the primary cause of the decomposition of the salt of copper, it is very easy to explain the subsequent phenomena. The steel, according to M. Becquerel, is one of the elements of the pile, the acid of the salt of copper is carried to the positive, and the oxide to the negative pole. This, again, is decomposed—the oxygen goes to the iron, which is oxidized, and dissolved in the sulphuric acid, and the copper is precipitated on the steel, covers it entirely, and opposes as mentioned above, the disengagement of the gas. This, however, does not constitute all the action, for the sulphuric acid which M. Boutigny has elsewhere shown to be added merely to render the water a better conductor of the electric fluid, acts on the steel by causing the decomposition of the water, the oxygen of which goes to the iron, while the hydrogen is evolved. The oxide of iron thus formed is dissolved in the sulphuric acid, and forms sulphate of iron, which remains in solution.

To insure the success of the experiment, the following conditions are necessary: 1*st*, a small bar of steel; 2*d*, an acid capable of dissolving the deutoxide of copper, and one of the elements of the steel, after being oxidized at the expense of the water, or of the oxide of copper, and having no action on metallic copper—sulphuric acid, fulfilling all these conditions, ought consequently to be preferred. The other conditions are a liquid, slightly, if at all, viscid, and of the same or nearly the same density as distilled water.

After the appearance of the phenomena enumerated above, there occurs a moment when they cease, which is known by the absence of bubbles, and most frequently by the horizontal position of the cylinder, which has replaced the needle on the surface of the liquid. This cylinder, being carefully lifted, and mixed with a grain of borax and a sufficient quantity of oil to form a paste, is to be placed in a testing-tube, and submitted to the action of a blowpipe, till it acquires a very deep red-brown color. In this state, the tube, when examined by the aid of a good magnifying glass will exhibit several points of metallic copper scattered over its surface.

The results of the facts and experiments narrated by M. Boutigny are, 1*st*, that articles of food and drink prepared in copper vessels, contain almost always more or less of that metal—a fact which renders it desirable that for this metal some other should be substituted; 2*d*, that wine, cider, and wheat, conceal sometimes atoms of this metal, but only when it is contained in the soil in which the vines, apple trees, and wheat grow; from which we may infer, that presence of copper in vegetables is *not the result of the act of vegetation*, but only of absorption; 3*d*, that the discovery of copper in articles of food a•d drink raises a question in legal medicine, which renders new investigations necessary, and which, in the meantime, ought to render experiments very circumspect in cases of poisoning by copper.—*Ed. Med. and Surg. Journ. from Journ. de Chimie. Med. March*, 1833, *from Am. Journ.*

17. *On the Transmission of Medicaments into the System by Means of Electro-Galvanism.*—It was stated some time since in one of the English journals, that by the influence of electricity the vaccine virus had been conducted along a wire, and the disease communicated from one person to another as effectually as when vaccination is performed in the ordinary manner. This statmement strongly excited our interest at the time, and we should have then called the attention of our readers to it had it not been coupled with the startling assertain that *intermittent fever*, during the hot stage, had been communicated by the same means to a perfectly healthy person. This last assertion evidently proved too much, and so completely destroyed all the confidence we were disposed to repose in the first, that it appeared to us expedient to wait for some confirmation of the statement, rather than give currency to it by repeating what might prove to be only one of the thousand baseless assertions constantly put forth by careless observers.

It appears from an editorial article in a late number of the *Gazatte Medicale de Paris*, for July 20th, 1833, that Dr. Fabré-Palaprat, previously to the publication of the statements just alluded to, had communicated to one of the Parasian societies a mass of observations relave to the property which he has found the electric fluid to possess, of conducting through our organs a number of active medicaments. It is known that electricity given off from a galvanic pile, has the power of decomposing most bodies, and of transferring their elements to one or other of its poles. This principle or power is the basis of the observations about to be detailed. Dr. Fabré-Palaprat, to whom science is indebted for numerous interesting experiments upon this subject, when he wishes to transfer a medicinal substance to any organ makes use of the voltaic pile, to each pole of which conductors are attached, which serve to transmit the electric current, and at the same time to decompose the substance upon which this current acts. One of the poles is made to communicate with the substance we wish to transfer, the other with a needle, (formed like that used in acupunctuation, of steel, silver, or platina, and of the same shape,) which is inserted into the part of organ to which we wish the medicine applied; for example, the thyroid gland or stomach. In a short time the medicine, let us suppose it to be *iodine* or *quinine*, passes with great rapidity, by some unknown route, to that part of the thyroid gland or stomach which is in communication with the point of the needle. This may be repeated, or the operation prolonged until a sufficient quantity of the iodine or quinine has been applied to the part. In order to prove that this transmission of the substance takes place, cases of goitre, and intermittent fever are cited, in which a cure of the disease took place from the operation. But it might be said, that in these cases a cure was due, not to the application of the medicine alone, but to other circumstances; as for instance, to the electro galvanism employed, as this is known to exercise a powerful influence over the animal economy! In order to set at rest this point, Dr. F. P. in conjunction with the editor of the *Gazatte Medicale*, undertook a series of experiments. The following is the manner in which they proceeded. Having prepared a vase of porcelain filled with a soultion of the hydriodate of potash, a platina thread was so arranged that whilst one end was plunged in the liquid, the other communicated with the copper pole of a pile of fifty large plates. Another wire of the same metal was made to communicate by one of its ends with the zinc pole of the pile, and by the other with a solution of starch contained in a vase similar to the first. Finally; in order to complete the circle, a finger of the right hand of the operator, was plunged into the iodine solution, and one of the left placed in contact with the platina plate. (We should observe that here, that portion of the conducting circle which passed from the body of the operator to the pile was perfectly dry.) In a few seconds after the circle was formed, in the manner described, several points of a violet tint were observed to form upon the thread which communicated with the starch. These points gradually enlarged, and after a time united one with another, until a complete line of violet hue was formed from the end of the thread to the hand. The appearance of this peculiar color, (violet,) left no doubt whatever as regards the translation of the iodine from the extremity of the conductor attached to the copper pole, to that of the zinc. The following is the chemical explanation of this phenomenon; at the moment that two electric currents disengaged from the two poles of the pile meet, the hydriodate of potash placed in contact with the copper pole is decomposed; the hydrogen is given off; the potash becomes dissolved in the water of the solution, and the iodine powerfully attracted by the positive current, passes rapidly to the zinc pole, as is shown by the violet color of the solution of starch, (the best test for iodine,) placed in communication with this pole. The iodine was selected in these experiments, on account of its presence in extremely minute portions even, being so readily discovered by a solution of starch. Reasoning from analogy, however, there can be no doubt, but that a similar translation of any other body, subject to the action of electricity, would also take place, provided we took the precaution of placing the organ to receive it in communication with that pole of the pile, towards which the electro-chemical disposition of the substance would cause it to proceed. We here repeated these experiments with the iodine, and failed in producing similar effects, notwithstanding every thing seemed to be arranged as it should be. Dr. F. P. has also frequently met with the same results. This failure, however, is but temporary, for with a little patience, and by frequently repeating the operation, we are generally rewarded with success. Dr. F. P. attributes this irregularity of action to the impermeability of the skin of the operator during the operation. If, as he supposes this irregularity is dependent upon the impermeable nature of the cutaneous tissue of the operator, it is evident that by the employment of metallic needles, this irregularity could not take place, for the conducting circle is here formed of inert matter; the conducting power of which is not affected by any accidental circumstance. However this may be, there rests not a doubt of the posibility of causing the translation of certain remedies to our organs through the medium of an electro-galvanic current, although we are as yet entirely ignorant of the route of this transmission, as well as of the mechanism of the causes which put it in play. Before closing this notice it may be as well to mention certain precautions which we should take in order to insure the success of the operation. In order that decomposition may take place, it is first necessary that the pile be possessed of a certain degree of energy, and further, that the electric current pass uninterruptedly through the different pieces of which the pile consists. These two indications are fulfilled, the first by multiplying with the number and the surface of the metallic parts of which the pile is formed, (of course its entire force will be in proportion to the number of the plates and the extent of their surfaces;) the second by interposing between the plates some body which is a good conductor of electricity. Water for instance, or what is still better, water containing a certain quantity of some salt or acid in solution. Dr. Fabré-Palaprat has also observed, that the decomposed bodies were conducted along a moist conductor only, so that if a part of the conductor of the pile be dry, and the remainder sufficiently moist, the substance experimented upon does not traverse the former, but only the latter. It may be remarked en passant, that as all those portions of the conducting circle, formed either by the needles or by the skin alone, are supposed to be perfectly dry; that there would be but little possibility of the substance operated with, passing in the direction of this portion; for as we have mentioned, this transmission takes pace only when the conductor is moist. But it is positive that the electric current circulates through the different organs into which the needles are inserted, as far even as the extremities of the needles. We may account for the occurrence of this phenomena in the following manner. The whole tract of the needles is imbued with a sufficient degree of moisture, derived from the fluids which circulate in all the tissues, and which are saline or alkeline in their nature, to cause an attraction of the electric current towards them. This moisture, moreover, from its alkaline properties is one of the most powerful conductors we could possibly make use of. Whenever we wish to operate with a substance of a compound nature, an acid for example, we should make use of a pile of feeble energy, that is to say one composed of but few plates, and these of a small size. If we use a powerful one the substance will be decomposed, and we transmit not the substance itself, but its elements. We may operate in this way when we wish to destroy by degress an exuberant tissue, by means of the nitrate of potash. A simple current would be sufficient to disengage the acid from its base, and cause it to pass over to the part operated upon. Another inconvenience likewise attends the application of too powerful a current. The quantity of electricity that passes over in this case, may be sufficient to communicate a fatal shock to the part; and if needles are used, their extremities may be heated to incandescence, and thus cauterize more or less deeply the parts into which they are inserted. Dr. Fabré-Palaprat, aware of this, has frequently applied a strong electric current to the needles inserted into a part in which he wished to produce the effects of a moxa.—*American Journal.*

18. *Weight of Man at different Ages.*—From observations made by M. Quetelet, on sixty-three male, and fifty-six female new-born infants, in the Maternité de Saint Pierre, at Brussels, it appears that the mean weight of the former was 3.20 kilogrammes, (6.536lbs,) while the length, by Chaussier's mecometer, was 0.496 metres, (1 foot, 6 inches, 3 lines;) and of the females, the mean weight was 2.91 kilog. (5.923lbs,) the length 0.483 metres, (1 foot, 5 inches, 10 lines.) Whence it is inferred that at birth there is an inequality in the weight and size of the two sexes—the males having the advantage in both.

Chaussier seems to have been the first who remarked that the infant, presently after birth, begins to lose some of its weight. M. Quetelet, from seven series of observations, extending in each case to the seventh day, has confirmed M. Chaussier's remark, and shows that the infant does not begin to grow perceptibly till after the first week.

M. Quetelet gives a table of the corresponding weights and statures at the different ages. We extract a few of them, by way of specimen:—

| Ages. | Males. | | Females. | |
|---|---|---|---|---|
| | Height. | Weight. | Height. | Weight. |
| | m. | k. | m. | k. |
| At Birth | 0.500 | 3.20 | 0.490 | 2.91 |
| 1 | 0.698 | 9.45 | 0.690 | 8.79 |
| 3 | 0.864 | 12.47 | 0.852 | 11.79 |
| 6 | 1.047 | 17.24 | 1.031 | 16.00 |
| 10 | 1.275 | 24.52 | 1.248 | 23.52 |
| 20 | 1.674 | 60.06 | 1.572 | 52.28 |
| 30 | 1.684 | 63.65 | 1.579 | 54.33 |
| 40 | 1.684 | 63.67 | 1.579 | 55.23 |
| 50 | 1.674 | 63.46 | 1.537 | 56.16 |
| 70 | 1.623 | 59.52 | 1.514 | 51.52 |

In order to render these results still more striking, the author has delineated, by two curves, the course which the weight takes in either sex. The ordinates of the curves expressing the weights, and the abscisses the ages. It is thus seen at a glance, that at any given period the man is generally heavier than the woman. About the age of twelve, however, it may be observed that the weights of both sexes are generally equal. This circumstance must, of course, be imputed to the earlier approach of puberty in the female.

Some of the other conclusions of M. Quetelet are curious. Man attains his maximum weight about the age of 40, and begins to lose weight very sensibly towards 60. Woman is not at her maximum weight till towards 50. Between 18 and 40, the period of her fecundity, she does not acquire any very perceptible increase of weight.

Both man and woman, at the period of their complete development, weigh almost exactly twenty times as much as they did at birth. Their height at the same period is about 3¼ times what it was at birth.

In their old age, both man and woman have lost 6 or 7 kilogrammes of their weight, and 7 centimetres of their height.

During the growth of both sexes, it may be stated that the squares of the weights are as the fifth powers of the heights.

After the development is complete in both sexes, the weights are very nearly as the squares of the heights: whence it may be inferred that the increase in the height is greater than the transverse increase of the body, comprehending both its breadth and depth.

The mean weight of an individual, without reference to sex or age, is 44.7 kilogrammes, (91.336lbs:) or, if sex be taken into account, it is 47 kilogrammes, (96.015lbs:) for men, and 42.5 kilogrammes, (86.-831bls.) for woman.

According to the observations of the late M. Tennon, of the Institute, (which observations are given by way of supplement to M. Quetelet's paper,) the Laplanders and the Patagonians present the two extremes of man's stature. The former commonly measure from 4 feet to 4 feet 6 inches, 4 feet three being their mean height, and their women are scarcely less. The Patagonians measure from about 5 feet 6 inches to 6 feet 3, and their women are generally 7 or 8 inches shorter.

The tallest men in Europe, M. Tenon thought, were in Saxony. But he added, that the climate or locality had less to do with the stature of men than their race or variety. Close by the Saxons, for example, we find the Silesians, who are a short people; and the Patagonians are the Pecherais, a people much inferior in height. In Savoy also, and particularly about *La Haute-Maurienne*, extreme varieties have been noticed. Yet it can scarcely be doubted but that the climate, the nature of the soil, the sort of government, the state of civilization, and the comforts, or the contrary, of each people, have much influence in determining a national stature. This position has been strongly urged by M. Villermé, in his *Memoir sur de l' Homme;* and very interestingly stated by W. F. Edwards, in his *Caracteres Physiologiques des Races.—Lond. Med. Gaz. September*, 1833, *from Am. Journal.*

19. *Lunatics in New-York.*—At a late meeting of the New-York Medical Society, the subject of lunacy was under consideration. It was ascertained that the number of lunatics and idiots in the State amounted to about *three thousand*, that about three-fourths of these are poor, and that the Asylum at Bloomingdale, and the private retreat of Dr. White of Hudson, can accomodate at most but three hundred—thus leaving about two thousand seven hundred of these wretched beings, in that single State, almost entirely destitute of medical or moral treatment, confined in poorhouses or jails, or private abodes, or wandering about the country, and treated, too often, more like brutes than human beings. This is indeed a melancholy picture; and when we refer to the records of our well-managed asylums, and see how all are treated with kindness, and how many have their reason restored to them, we cannot but believe that the Legislature of New York will respond cheerfully and speedily to the call of the Society for money to erect a suitable edifice for the reception and treatment of these unfortunate beings.

The Legislature of the State of Maine has established, at its recent setting, a noble example in this species of benificence. It has appropriated 20,000 dollars for the establishment of an asylum for the insane, on the condition that other 20,000 dollars shall be raised by private subscription. A single individual in that State promptly sub scribed half this sum, and no doubt can exist that the remainder will be speedily obtained.

Probably there is no department of science, no form of humanity, in which greater advances have been made of late years, than in the medical and moral management of the insane. When we contrast the spacious and airy apartments and grounds of our asylums, with the dark, and narrow, and dirty cells, in which, twenty years ago, the best accommodated of these poor creatures were immured—their neat and comfortable dress, with their former rags and nakedness—their wholesome food, with their former rations—and above all, the kindness and affection which is shown to them now, with their utter neglect in the days when they were excluded from the privileges and the society of men, we find ourselves shuddering at the thought of what we *have seen*, and lost in admiration of what we *now see*.

Wherever the christian religion exists, we find the same rapid advances making towards the accomplishment of the great purposes of humanity. It seems as if the miracles of our Saviour were meant as prototypes of what his religion was to accomplish. It is by the influence of this religion on the march of science and philosophical discovery, that, by all christian nations, the winds and the waves have been rebuked—that man is enabled to ride out the storm upon the ocean, as if it were hushed, and, like Peter of old, to walk upon the sea as on dry land. Institutions, too, throughout christendom, are eitheir already established or fast rising up, where the blessings of sight are bestowed upon the blind, and of hearing upon the deaf;—where the dumb are made to speak—lepers are cleansed, the sick are healed, the lame are made to walk, the maimed restored to their integrity, and where evil spirits even are cast out, and the poor lunatic receives back again his lost reason.

Among the improvements in the mode of managing the insane, that have been recently adopted with marked success, we have been particularly impressed with the wisdom of the plan which furnishes them *employment*. Among the numerous inmates of our asylums, we apprehend there are few who have always been accustomed to regular bodily labor, and the degree of mental action that is usually required by occupations of this description. Employment is the door that keeps health in the body and reason in its proper abode. Take it away, and the one will certainly take its flight, and the other be not unlikely to follow. It seems rational, therefore, that when the means resorted to in the treatment of the insane, have, to a certain degree, been successful, the furnishing such occupation as has a plain and palpable object is a wise and promising measure. A familiar account of its adoption in Europe will be found in the following extract from one of the letters of Mr. Willis.

"Two of the best conducted lunatic asylums," says he, "in the world, are in the kingdom of Naples—one at Aversa, near Capua, and the other at Palermo. The latter is managed by a whimsical Sicilian baron, who has devoted his time and fortune to it, and, with the assistance of the Government, has carried it to a great extent and perfection. The poor are received gratuitously; and those who can afford it, enter as boarders, and are furnished with luxuries according to their means.

The hospital stands in an airy situation in the lovely neighborhood of Palermo. We were received by a porter in a respectable livery, who introduced us immediately to the old baron—a kind-looking man, rather advanced beyond middle life, of manners singularly mild and prepossessing. '*Je suis le premier fou,*' said he, throwing his arms out. as he bowed on our entrance. We stood in an open court, surrounded with porticoes and lined with stone seats. On one side of them lay a fat indolent-looking man, in clean grey clothes, talking to himself with great apparent satisfaction. He smiled at the baron as he passed, without checking the motion of his lips, and three others standing in the door-way of a room marked as the kitchen, smiled also as he came up, and fell into his train, apparently as much interested as ourselves in the old man's explanation.

The kitchen was occupied by eight or ten people all at work, and all, the baron assured us, mad. One man of about forty, was broiling a steak with the gravest attention. Another who had been furious till employment was given him, was chopping meat with violent industry in a large wooden bowl. Two or three girls were about obeying the little orders of a middle-aged man, occupied with several messes cooking on a patent stove. I was rather incredulous about his insanity, till he took a small bucket and went to the jet of a fountain, and getting impatient from some cause or other, dashed the water upon the floor. The baron mildly called him by name, and mentioned to him, as a piece of information, that he had wet the floor. He nodded his head, and filling his bucket quietly, poured a little into one of the pans, and resumed his occupation.

We passed from the kitchen into an open court, curiously paved, and ornamented with Chinese grottoes, artificial rocks, trees, cottages and fountains. Within the grottoes reclined figures of wax. Before the altar of one, fitted up as a Chinese chapel, a mandarin was prostrated in prayer. The walls on every side were painted in prospective scenery, and the whole had as little the air of a prison as the open valley itself. In one of the corners was an unfinished grotto, and a handsome young man was entirely absorbed in thatching the ceiling with strips of cane. The baron pointed to him and said he had been incurable till he had found this employment for him. Everything about us, too, he assured us, was the work of his patients. They had paved the court, built the grottoes and cottages, and painted the walls under his direction. The secret of his whole system, he said, was employment and constant kindness. He had usually about one hundred and fifty patients, and he dismissed upon an average two-thirds of them quite recovered.

We went into the apartment of the women. These, he said, were his worst subjects. In the first room sat eight or ten employed in spinning, while one infuriated creature, not more than thirty, but quite gray, was walking up and down the floor, talking and gesticulating with the greatest violence. A young girl of sixteen, an attendant had entered into her humor, and with her arm put affectionately round her waste, assented to every thing she said, and called her by every name of endearment while endeavoring to silence her. When the Baron entered, the poor creature addressed herself to him, and seemed delighted that he had come. He made several mild attempts to check her, but she seized his hands, and with the veins of her throat swelling with passion, her eyes glaring terribly, and her tongue white and trembling she continued to declaim more and more violently. The baron gave an order to a male attendant at the door, and beckoned us to follow, led her gently through a small court planted with trees, to a room containing a hammock. She checked her torrent of language as she observed the preparations going on, and seemed amused with the idea of swinging. The man took her up in his arms without resistance, and laced the hammock over her, confining every thing but her head, and the female attendant, one of the most playful and prepossessing little creatures I ever saw, stood on a chair, and at every swing threw a little water on her face as if in sport. Once or twice the maniac attempted to resume the subject of her ravings, but the girl laughed in her face and diverted her from it, till at last she smiled, and dropping her head into the hammock, seemed disposed to sink into an easy sleep.

We left her swinging, and went out into the court, where eight or ten women, in the gray gowns of the establishment, were walking up and down, or sitting under the trees, lost in thought. One, with a fine intelligent face, came up to me and curtsied gracefully without speaking. The physician of the establishment joined me at the moment, and asked her what she wished. 'To kiss his hand,' said she, 'but his looks forbade me.' She colored deeply, and folding her arms across her breast, walked away. The baron called us, and in going out I passed her again, and taking her hand, kissed it and bade her good-by. 'You had better kiss my lips,' said she, 'you will never see me again.' She laid her forehead against the iron bars of the gate, and with a face working with emotion, watched us till we turned out of sight. I asked the physician for her history. 'It was a common case,' he said. 'She was the daughter of a Sicilian noble, who, too poor to marry her to one of her own rank, had sent her to a convent, where confinement had driven her mad. She is now a charity patient in the asylum.'

The courts in which these poor creatures were confined, opened upon a large and lovely garden. We walked through it with the baron, and then returned to the apartments of the females. In passing a cell, a large majestic woman, striding out with a theatrical air, commenced an addres to the Deity, in a language strangely mingled of Italian and Greek. Her eyes were naturally large and soft, but excitement had given them additional dilation and fire, and she looked a prophetess. Her action, with all its energy, was lady-like. Her feet, half covered with slippers, were well formed and slight, and she had every mark of superiority both of birth and endowment. The baron took her by the hand with the deferential courtesy of the old school, and led her to one of the stone seats. She yielded to him politely, but resumed her harangue, upbraiding the Deity, as well as I could understand her, for her misfortunes. They succeeded in soothing her by the assistance of the same playful attendant who had accompanied the other to the hammock, and she sat still, with her lips white and her tongue trembling like an aspen. While the good old man was endeavoring to draw her into a quiet conversation, the physician told me some curious circumstances respecting her. She was a Greek, and had been brought to Palermo when a girl. Her mind had been destroyed by an illness, and after seven years' madness, during which she had refused to rise from her bed, and had quite lost the use of her limbs, she was brought to this establishment by her friends. Experiments were tried in vain to induce her to move from her painful position. At last the baron determined upon addressing what he considered the master passion in all female bosoms. He dressed himself in the gayest manner, and, in one of her gentler moments, entered the room with respectful ceremony, and offered himself to her in marriage! She refused him with scorn, and with seeming emotion he begged forgiveness and left her. The next morning, on his entrance she smiled—the first time for years. He continued his attentions for a day or two, and after a little coquetry, she one morning announced to him that she had reconsidered his proposal, and would be his bride. They raised her from her bed to prepare her for the ceremony, and she was carried in a chair to the garden, where the bridal feast was spread, nearly all the other patients of the asylum being present. The gaiety of the scene absorbed the attention of all; the utmost decorum prevailed; and when the ceremony was performed, the bride was crowned, and carried back in state to her apartment. She recovered gradually the use of her limbs, her health is improved, and excepting an occasional paroxysm, such as we happened to witness, she is quiet and contented. The older inmates of the asylum still call her the bride; and the baron, as her husband, has the greatest influence over her.

While the physician was telling me these circumstances, the baron had succeeded in calming her, and she sat with her arms folded, dignified and silent. He was still holding her hand, when the woman whom we had left swinging in the hammock, came stealing up behind the trees on tiptoe, and putting her hand suddenly over the baron's eyes, kissed him on both sides of his face, laughing heartily, and calling him by every name of affection. The contrast between this mood and the infuriated one in which we had found her, was the best comment on the good man's system. He gently disengaged himself, and apologized to his lady for allowing the liberty, and we followed him to another apartment. It opened upon a pretty court, in which a fountain was playing, and against the different columns of the portico sat some half dozen patients. A young man of eighteen, with a very pale scholar-like face, was reading Ariosto. Near him, under the direction of an attendant, a fair, delicate girl, with a sadness in her soft blue eyes that might have been a study for a *mater dorolosa*, was cutting paste upon a board laid across her lap. She seemed scarcely conscious of what she was about, and when I approached and spoke to her, she laid down her knife and rested her head upon her hand, and looked at me steadily, as if she was trying to recollect where she had known me. 'I cannot remember,' said she to herself, and went on with her occupation. I bowed to her as we took our leave, and she returned it gracefully but coldly. The young man looked up from his book and smiled, the old man lying on the stone seat in the outer court rose up and followed us to the door, and we were bowed out by the baron and his genteel madmen as politely and kindly as if we were concluding a visit to a company of friends."—*Boston Medical and Surgical Journal.*

